# Epigenetic upregulation of ARL4C, due to DNA hypomethylation in the 3'-untranslated region, promotes tumorigenesis of lung squamous cell carcinoma

**DOI:** 10.18632/oncotarget.13147

**Published:** 2016-11-07

**Authors:** Shinsuke Fujii, Keiko Shinjo, Shinji Matsumoto, Takeshi Harada, Satoshi Nojima, Sunao Sato, Yu Usami, Satoru Toyosawa, Eiichi Morii, Yutaka Kondo, Akira Kikuchi

**Affiliations:** ^1^ Department of Molecular Biology and Biochemistry, Graduate School of Medicine, Osaka University, Suita 565-0871, Japan; ^2^ Department of Epigenomics, Nagoya City University Graduate School of Medical Sciences, Kawasumi, Mizuho-cho, Mizuho-ku, Nagoya 467-8601, Japan; ^3^ Department of Pathology, Graduate School of Medicine, Osaka University, Suita 565-0871, Japan; ^4^ Department of Oral Pathology, Graduate School of Dentistry, Osaka University, Suita 565-0871, Japan; ^5^ Present address: Laboratory of Oral Pathology, Division of Maxillofacial Diagnostic and Surgical Sciences, Faculty of Dental Science, Kyushu University, Higashi-ku, Fukuoka 812-8582, Japan

**Keywords:** ARL4C, DNA methylation, CpG islands, tissue differential methylation region, lung cancer

## Abstract

ADP-ribosylation factor (ARF)-like 4c (ARL4C) expression, induced by a combination of Wnt/β-catenin and EGF/Ras signaling, has been demonstrated to form epithelial morphogenesis. ARL4C overexpression, due to Wnt/β-catenin and EGF/Ras signaling alterations, was involved in tumorigenesis. It was also reported that ARL4C expression correlates with DNA hypomethylation in the 3’-untranslated region (UTR) of *ARL4C* gene during lymphogenesis. The current study was conducted to investigate the expression and functions of ARL4C due to DNA hypomethylation in lung and tongue cancers. Immunohistochemical analyses of tissue specimens obtained from lung and tongue squamous cell carcinoma (SCC) patients revealed that ARL4C is not observed in non-tumor regions, but is strongly expressed at high frequencies in tumor lesions. Although inhibition of Wnt/β-catenin or Ras/MAP kinase signaling did not decrease ARL4C expression in NCI-H520 lung SCC cells, *ARL4C* DNA was clearly hypomethylated in the 3’-UTR. Ten-eleven translocation methylcytosine dioxygenase (TET) enzyme, which mediates DNA demethylation, was highly expressed in NCI-H520 cells. Knockout of TET family proteins (TET1-3) in NCI-H520 cells reduced 5-hydroxymethylcytosine (5hmC) levels and promoted DNA methylation in the 3’-UTR, leading to the decrease in ARL4C expression and ARL4C-mediated cellular migration. In tumor lesions of ARL4C-positive lung SCC, 5hmC was frequently detected and DNA methylation in the 3’-UTR of *ARL4C* gene was lower than in non-tumor regions, which were consistent with the Cancer Genome Atlas dataset. These results suggest that ARL4C is expressed due to hypomethylation in the 3’-UTR for certain types of cancers and that *ARL4C* methylation status is involved in cancer cell function.

## INTRODUCTION

ADP-ribosylation factor (ARF)-like (Arl) is one of the eight members of the Arf subfamily, which belong to the small GTP-binding superfamily [1, 2]. ARL4C was isolated by 5’-RACE after searching the EST database [3]. ARL4C has been reported to stimulate cholesterol efflux in HeLa cells [4] and to be involved in transport of transferrin from early endosomes to recycling endosomes by interacting with α-tubulin in human renal carcinoma cells [5], but the detailed modes of expression and action of ARL4C are not well understood. A recent study revealed that ARL4C changes cell morphology and stimulates cellular migration through the regulation of the small G proteins, Arf6, Rac1, and RhoA, as one of mode of action, and stimulates cellular migration and promotes cellular proliferation through YAP, thereby forming tube-like structures of cultured epithelial cells and uretic bud formation in the embryonic kidney [6]. As a mode of expression, it has been shown that Ets, a mitogen-activated protein kinase (MAPK) signal transcriptional factor, constitutively binds to the 3’-untranslated region (3’-UTR) of the *ARL4C* gene, where it forms a complex with β-catenin and a Wnt signaling pathway transcription factor Tcf4, in response to exposure to a combination of Wnt3a and EGF, thereby inducing *ARL4C* mRNA expression through enhancement of histone H3 acetylation [6].

Genetic alterations of Wnt/β-catenin and EGF/Ras pathways are common in various types of cancer [7]. ARL4C was indeed highly expressed in tumor lesions of colon and lung adenocarcinomas, and ARL4C expression promoted migration, invasion, and proliferation of cancer cells both *in vitro* and *in vivo* [8]. Furthermore, since ARL4C knockdown by *in vivo* siRNA suppressed xenograft tumor formation, ARL4C might represent a novel therapeutic target for cancers with ARL4C overexpression [8].

It is generally accepted that tumor cell genomes are hypomethylated relative to non-tumor counterparts but show gene-specific hypermethylation [9]. The underlying cause of genome-wide hypomethylation in cancers remains unknown, but the hypomethylation might cause genome instability and reactivation of transposons, resulting in the aberrant activation of oncogenes. Aberrant hypermethylation in cancer usually occurs at CpG islands, and the resulting changes effectively suppress transcription of tumor suppressor genes [10]. In contrast, oncogene expression due to gene-specific hypomethylation also occurs in cancer. For example, the S100 calcium binding protein A4 (S100A4) gene, which is known as a metastasis-associated gene, is frequently demethylated and its protein expression is increased in colon and pancreatic cancers [11, 12]. Demethylation accompanied by increased expression was also reported for maspin, the serine protease inhibitor, in gastric cancer [13], the putative oncogene γ-synuclein (SNCG) in breast and ovarian cancers [14], and Wnt5a in prostate cancer [15]. Thus, alterations in DNA methylation occur in cancer, including hypermethylation of tumor suppressor genes and hypomethylation of oncogenes.

Recently, several studies concerning the alternation in DNA methylation status on tumorigenesis in lung cancers, especially in non-small cell lung cancer (NSCLC), have been reported. For instance, DNA methylation was associated with aberrant gene expression, leading to tumorigenesis in NSCLC, such as squamous cell carcinoma (SCC) [16]. DNA methyltransferases (DNMTs) were highly expressed and its expression was associated with poor prognosis in NSCLC patients [17–20]. In addition, the methylation status was inversely correlated with gene expression, such as *CDKN2A*, *AXL*, *HOB4*, *MST1R* in NSCLC [21]. Hypermethylation of the promoter of tumor suppressor genes, such as *FHIT*, *p16^INK4a^* and *RARβ*, was correlated with high expression of DNMT1 in NSCLC [19]. Although these findings suggest that NSCLC tumorigenesis is correlated with down regulation of tumor suppressor genes through DNA hypermethylation, methylation status of oncogenes or functional roles of Ten-eleven translocation methylcytosine dioxygenases (TET1, TET2, and TET3), which convert 5-methylcytosine (5mC) to 5-hydroxymethylcytosine (5hmC), in tumorigenesis are unclear.

Alteration of DNA methylation in promoters and CpG islands is functionally important for gene transcription [10]. However, comprehensive high-throughput array-based relative methylation analysis revealed that most tissue-specific methylation occurs at tissue differential methylation regions (T-DMRs), most of which are located near CpG islands (in sequence up to 2 kb distant from transcription starting site)[22]. The study also showed that most cancer-related DNA methylation regions, at least in colon cancer, overlap T-DMRs, suggesting that DNA methylation alterations occur around T-DMRs in cancer.

In hematopoiesis, multipotent progenitors strictly differentiate into myeloid or lymphoid progenitors, including common lymphoid progenitors, common myeloid progenitors, granulocyte/macrophage progenitors (GMPs), and thymocyte progenitors (DN1, DN2, and DN3)[23]. It was shown that the *ARL4C* gene is hypomethylated in T-DMRs in DN1-3 thymocytes and expression is upregulated [24], suggesting that ARL4C expression is involved in lymphogenesis. These results prompted us to examine *ARL4C* DNA methylation in cancer. Here we show that in lung SCCs *ARL4C* DNA is hypomethylated in the 3’-UTR, which corresponds to hypomethylation sites during lymphogenesis, rather than the promoter region. We also find that the TET is implicated in the *ARL4C* DNA methylation state.

## RESULTS

### Expression of ARL4C in squamous cell carcinomas

**Figure 1:**
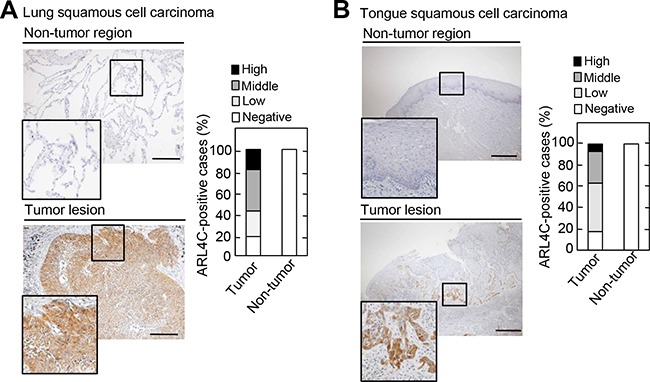
ARL4C is expressed in human lung and tongue squamous cell carcinomas **A-B.** Lung squamous cell carcinoma tissues (n = 62) A. and tongue squamous cell carcinoma tissues (n = 57) B. were stained with anti-ARL4C antibody and hematoxylin. Percentages of ARL4C-positive cases in the tumor lesions and non-tumor regions are shown in the right panel. Areas staining positive for ARL4C are classified as follows: negative, < 5%; low, 5-20%; middle, 20-50%; high, 50-95%. Black boxes show enlarged images. Scale bars in A-B. are 200 μm.

Whether ARL4C is expressed in human cancers other than adenocarcinomas, such as colon and lung cancers, was investigated in SCCs. In lung SCCs, ARL4C was strongly detected in 50/62 (80.6%) of tumor lesions, while it was not detected in non-tumor regions (Figure 1A). The stained areas were classified into four categories (< 5%, 5-20%, 20-50%, and 50-95%) (Figure 1A and Supplementary Figure S1A), and the results were considered positive when the total area of a tumor lesion showed > 5% staining. The result of positive expression and the size of the area showing ARL4C expression were not correlated with the T grade (tumor size and depth of invasion) or N grade (degree of lymph node metastasis) of the tumor (Table 1), suggesting that ARL4C might be involved in the initiation, rather than progression, of lung SCCs.

**Table 1:** Relationship between ARL4C expression and clinicopathological characteristics of lung squamous cell carcinoma cases (n = 62)

	ARL4C		*P* value
	Positive	Negative	
T classification			
T1	20 (87%)	3	0.5082
T2/3	30 (76.9%)	9	
N classification			
N0	43 (81.1%)	10	0.6583
N1/2	7 (77.8%)	2	

Abbreviations: T1, Tumor greatest dimension is < 3 cm, surrounded by lung or visceral pleura, without bronchoscopic evidence of invasion more proximal than the lobar bronchus. T2, Tumor > 3 cm but < 7 cm or tumor with any of the following features: involves main bronchus, > 2 cm distal to the carina or invades visceral pleura or associated with atelectasis or obstructive pneumonitis that extends to the hilar region but does not involve the entire lung; T3, Tumor > 7 cm or one that directly invades any of the following: chest wall (including superior sulcus tumors), diaphragm, phrenic nerve, mediastinal pleura, parietal pericardium or tumor in the main bronchus < 2 cm distal to the carina but without involvement of the carina or associated atelectasis or obstructive pneumonitis of the entire lung or separate tumor nodule(s) in the same lobe.

N0, No regional lymph node metastasis. N1, Metastasis in ipsilateral, peribronchial and/or ipsilateral hilar lymph nodes and intrapulmonary nodes, including involvement by direct extension. N2, Metastasis in ipsilateral mediastinal and/or subcarinal lymph node(s).

ARL4C was also expressed in 42/57 (73.7%) of tumor lesions from tongue SCCs, but not in non-tumor regions of the tongue (Figure 1B and Supplementary Figure S1B). It is of note that ARL4C was strongly expressed in some cases from the advancing areas of tumor lesions, which are invading in the surrounding stroma. Tongue cancers tended to exhibit this staining pattern more than lung cancers. As compared to the level of *ARL4C* mRNA expression in HeLaS3 cervical cancer cells, *ARL4C* mRNA was highly expressed in NCI-H520 lung SCC cells and SAS tongue SCC cells (Supplementary Figure S1C).

### ARL4C is required for cell migration and proliferation of SCCs

**Figure 2:**
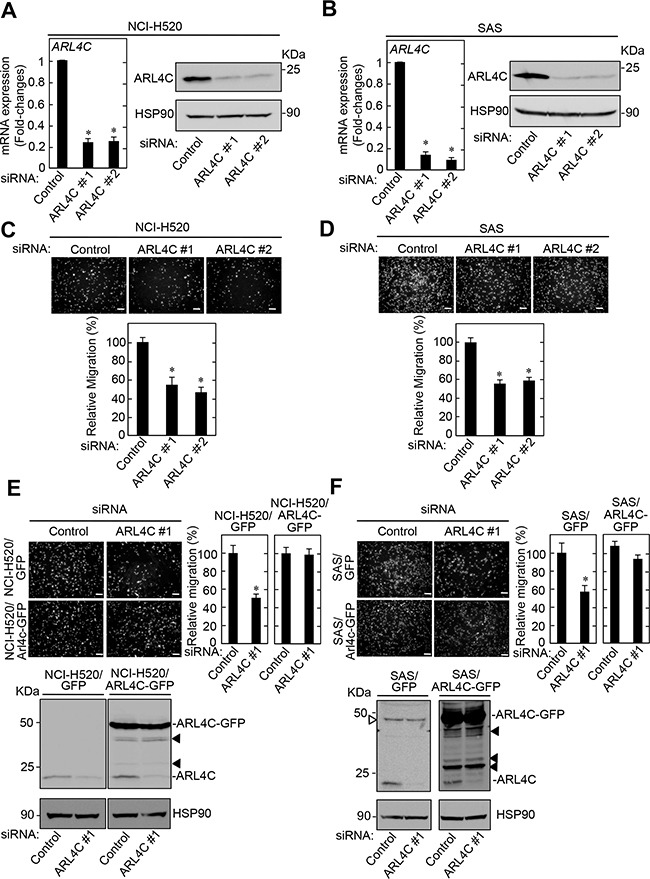
ARL4C knockdown inhibits migration of NCI-H520 and SAS cells **A-B**. NCI-H520 A. and SAS B. cells were transfected with control or two independent ARL4C siRNAs, and *ARL4C* mRNA levels were measured by quantitative RT-PCR. Relative *ARL4C* mRNA levels were normalized by *GAPDH* and expressed as fold-changes compared with levels in control siRNA transfected cells. Cell lysates were probed with anti-ARL4C and anti-HSP90 antibodies. **C-D**. NCI-H520 (C) and SAS (D) cells were transfected with control or two independent ARL4C siRNAs and then placed in Transwell chamber for the migration assay. Migration activities are expressed as the percentage of control cells. **E-F**. NCI-H520 cells stably expressing GFP or ARL4C-GFP (E) and SAS cells stably expressing GFP or ARL4C-GFP (F) were transfected with control or ARL4C siRNA #1 and then placed in Transwell chamber for the migration assay. Migration activities are expressed as the percentage of that seen in control cells. Cell lysates of NCI-H520 and SAS cells were probed with anti-ARL4C and anti-HSP90 antibodies. Closed triangles in E-F. indicate degradation products of exogenously expressed ARL4C-GFP and open triangle F. indicates a non-specific band. Scale bars in (C-F) are 200 μm. Results are shown as means ± s.d. of three independent experiments. ^*^, *P* < 0.01.

ARL4C expression was involved in migration and proliferation of HCT116 colon cancer and A549 lung adenocarcinoma cells [8]. To examine the roles of ARL4C in NCI-H520 and SAS cells, ARL4C was knocked down by two different siRNAs, which target the 3’-UTR (see Methods) (Figure 2A and 2B). ARL4C knockdown decreased migration of NCI-H520 and SAS cells, and ARL4C expression rescued the ARL4C-knockdown phenotype of cancer cells, excluding siRNA off target effects (Figure 2C-2F).

**Figure 3:**
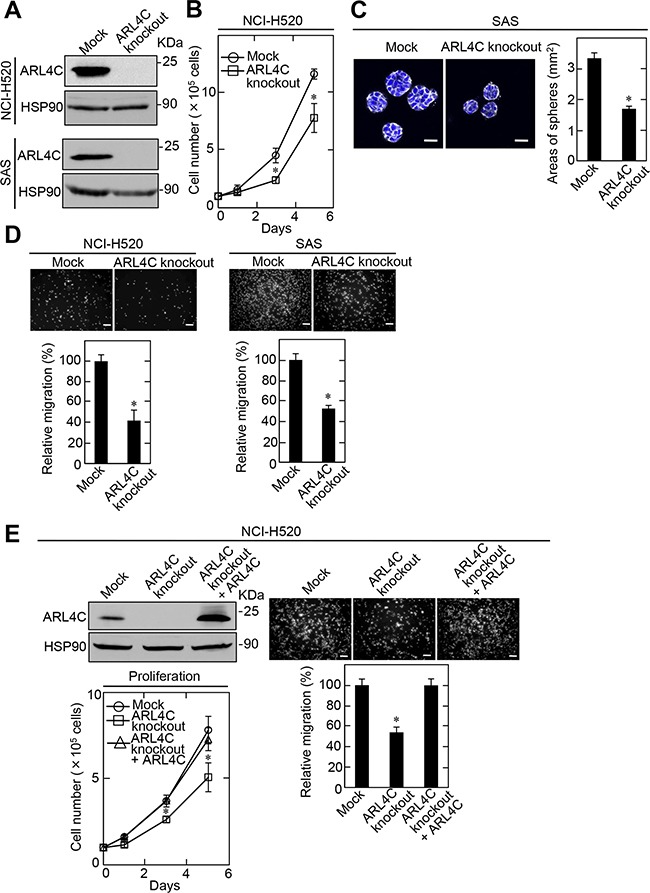
ARL4C knockout suppresses proliferation and migration of NCI-H520 and SAS cells **A.** Lysates of ARL4C knockout NCI-H520 (top) and SAS (bottom) cells were generated and cell lysates were probed with anti-ARL4C and anti-HSP90 antibodies. **B.** ARL4C control or knockout NCI-H520 cells were cultured on a two-dimensional (2D) plastic dish in the presence of 10% serum for the indicated numbers of days, and cell numbers were counted. **C.** ARL4C control or knockout SAS cells were cultured for 4 days in 3D Matrigel. The cells were then stained with phalloidin and DRAQ5, and the areas of spheres were calculated. **D.** ARL4C control or knockout NCI-H520 (left) and SAS (right) cells were placed in Transwell chamber for the migration assay. Migration activities are expressed as the percentage of control cells. **E.** ARL4C control or knockout NCI-H520 cells expressing mock or ARL4C for rescue experiments were cultured on a 2D plastic dish in the presence of 10% serum and placed in Transwell chamber for the migration assay. Cell numbers were counted and migration activities are expressed as the percentage of control cells. Cell lysates were probed with anti-ARL4C and anti-HSP90 antibodies. Results are shown as means ± s.d. of three independent experiments. ^*^, *P* < 0.01. Scale bars in C., 40 μm;in D-E., 200 μm.

Knockdown of ARL4C by shRNA did not affect SCC cell proliferation in two-dimensional (2D) culture conditions (data not shown). Therefore, ARL4C-knockout SCC cells were generated using a CRISPR/Cas9 system (Supplementary Figure S2A). Genomic deletion and the complete loss of ARL4C protein in the knockout cells were confirmed (Figure 3A and Supplementary Figure 2B). ARL4C knockout suppressed proliferation of NCI-H520 cells in 2D culture conditions (Figure 3B). When SAS cells were grown in 3D culture conditions with Matrigel, knockout of ARL4C reduced the spherical area of the tumor by half (Figure 3C). These results suggest that ARL4C is involved in tumor formation through proliferation and increasing invasive growth into stromal tissue. ARL4C knockout also decreased the migration activity of NCI-H520 and SAS cells (Figure 3D), while ARL4C expression rescued the ARL4C-knockout phenotype in NCI-H520 cells (Figure 3E), excluding off target effects of the ARL4C knockout by CRISPR/Cas9.

### Hypomethylation in the 3’-UTR is involved in ARL4C expression

**Figure 4:**
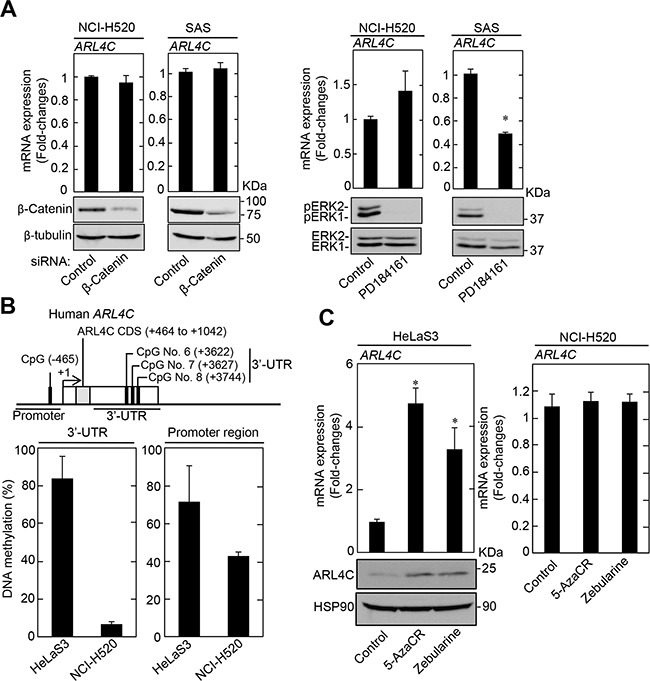
ARL4C expression in lung and tongue cancer cell lines **A.** Levels of *ARL4C* mRNA in NCI-H520 and SAS cells, which were transfected with β-catenin siRNA for 48 hours (left two graphs) or treated with 10 μM PD184161 for 24 hours (right two graphs), were measured and expressed as fold-changes compared with levels in NCI-H520 or SAS control cells, respectively. Cell lysates were probed with anti-β-catenin, anti-β-tubulin anti-phospho-ERK1/2 (pERK1/2, T202/Y204), or anti-ERK1/2 antibodies. **B.** *ARL4C* CpG sites that are analyzed for DNA methylation are indicated. DNA methylation status of *ARL4C* in HeLaS3 and NCI-H520 were examined by bisulfite pyrosequencing. DNA methylation levels of the 3’-UTR indicate means of those of CpG No. 6 to No. 8. **C.** HeLaS3 or NCI-H520 cells were treated with 50 μM 5-azacytidine (5-AzaCR) or 50 μM Zebularine for 5 days and *ARL4C* mRNA levels in the cells were measured by quantitative RT-PCR. Relative levels of *ARL4C* mRNA expression were normalized by *GAPDH* and expressed as fold-changes compared with expression in HeLaS3 or NCI-H520 control cells. Cell lysates were probed with anti-ARL4C and anti-HSP90 antibodies. Results are shown as means ± s.d. of three independent experiments. ^*^, *P* < 0.01.

It was shown that *ARL4C* mRNA is efficiently transcribed by the simultaneous activation of Wnt/β-catenin and EGF/Ras pathways and that inhibition of these pathways suppressed *ARL4C* mRNA expression [6]. However, knockdown of β-catenin did not affect *ARL4C* mRNA expression in NCI-H520 and SAS cells (Figure 4A). The inhibition of the MAPK pathway by PD184161, a MEK1/2 inhibitor, suppressed *ARL4C* mRNA expression in SAS cells but not in NCI-H520 cells (Figure 4A), suggesting that *ARL4C* mRNA expression was dependent upon Ras/MAPK signaling in SAS cells. However, *ARL4C* mRNA might be transcribed in NCI-H520 cells by a different way from HCT116 and A549 cells [8].

It was also reported that transcription of *ARL4C* mRNA is increased by demethylation in the T-DMRs, in which 3 (CpG No.6 to CpG No.8) of 8 CpGs were highly hypomethylated, of the *ARL4C* gene 3’-UTR during T-cell development [24]. Since the 3’-UTR (+2988 to +3744) of the human *ARL4C* gene also contains 8 CpGs, the methylation levels of CpG No. 6 (+3622), CpG No. 7 (+3627), and CpG No. 8 (+3744) were analyzed for the 3’-UTR of the *ARL4C* gene. The methylation status of CpGs in the promoter (-465) and the 3’-UTR of the *ARL4C* gene, as determined by bisulfite pyrosequencing in NCI-H520 cells, was lower than in HeLaS3 (Figure 4B). Consistent with these results, treatment of HeLaS3 cells with the DNA methylation inhibitors, 5-azacytidine (5-AzaCR) and Zebularine, increased the levels of ARL4C mRNA and protein, whereas these inhibitors did not affect *ARL4C* mRNA levels in NCI-H520 cells (Figure 4C).

### TET knockout suppresses ARL4C expression

**Figure 5:**
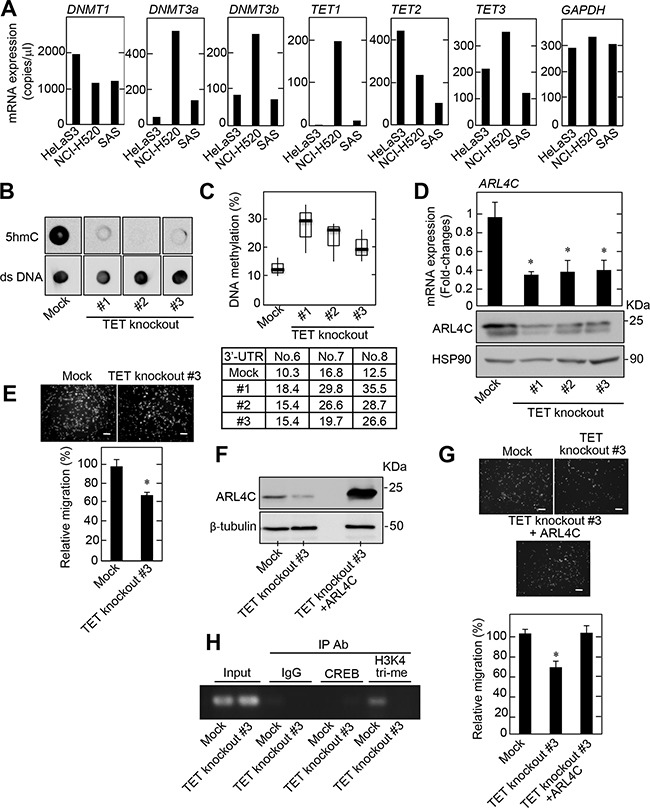
DNA methylation affects ARL4C expression **A.** *DNMT1, DNMT3a, DNMT3b*, *TET1, TET2, TET3,* and *GAPDH* mRNA levels in HeLaS3, NCI-H520, and SAS cells were measured by digital PCR and then copy numbers of these genes are shown. **B.** TET family knockout NCI-H520 cells were generated. Analysis of 5hmC levels in genomic DNA isolated from TET family knockout NCI-H520 cell clones was performed by dot blot assay using an anti-5hmC antibody. Anti-double strand (ds) DNA antibody was probed as a control. **C.** DNA methylation status of the *ARL4C* 3’-UTR in TET family knockout NCI-H520 cells was examined by bisulfite pyrosequencing. **D.** *ARL4C* mRNA levels in TET family knockout NCI-H520 cells were measured by quantitative RT-PCR and relative levels of *ARL4C* mRNA expression were normalized to *GAPDH* and expressed as fold-changes compared with control cells. Cell lysates were probed with anti-ARL4C and anti-HSP90 antibodies. **E.** Control or TET family knockout NCI-H520 cells were placed in Transwell chamber for the migration assay. Migration activities were expressed as the percentage of control cells. **F.** Cell lysates of control or TET family knockout NCI-H520 cells expressing mock or ARL4C were probed with anti-ARL4C and anti-β-tubulin antibodies. **G.** Control or TET family knockout NCI-H520 cells expressing mock or ARL4C were placed in Transwell chamber for the migration assays. Migration activities are expressed as the percentage of control cells. **H.** Chromatin from control or TET family knockout NCI-H520 cells was immunoprecipitated with anti-control IgG or anti-CREB or anti-histone H3 (tri methyl K4) antibodies and the precipitates were analyzed by PCR for *Arl4c* 3′-UTR (CpG No. 6 to No. 8.). Results are shown as means ± s.d. of three independent experiments. ^*^, *P* < 0.01. Scale bars in E and G are 200 μm.

Several enzymes, including DNMTs (DNMT1, DNMT3a, and DNMT3b) and TETs (TET1, TET2, and TET3) are involved in the regulation of DNA methylation [10, 25]. *DNMT1* mRNA levels were similar among HeLaS3, NCI-H520, and SAS cells when they were measured by digital PCR and quantitative PCR (Figure 5A and Supplementary Figure S3A). *DNMT3a* and *DNMT3b* were highly expressed in NCI-H520 cells (Figure 5A and Supplementary Figure S3A). As shown in Figure 4B, *ARL4C* DNA in NCI-H520 cells was hypomethylated. Exogenous expression of DNMT3a and DNMT3b did not affect *ARL4C* mRNA levels in NCI-H520 cells (Supplementary Figure S3B), and siRNAs against DNMT3a and DNMT3b did not affect *ARL4C* mRNA expression in HeLaS3 cells (Supplementary Figure S3C). These results suggest that altered ARL4C expression would not be directly regulated by DNMTs. Therefore, the effect of the TET proteins on *ARL4C* DNA methylation was examined.

The mRNA levels of the three TET isoforms were highly expressed in the NCI-H520 cells when they were measured by digital PCR and quantitative PCR (Figure 5A and Supplementary Figure S3A). Although *ARL4C* mRNA levels were slightly, but significantly, decreased by a using a combination of siRNAs against the three TET proteins (Supplementary Figure S4A), the slight reduction of *ARL4C* mRNA by knockdown of the three TET proteins did not affect NCI-H520 cell migration (Supplementary Figure S4B). Therefore, TET-knockout NCI-H520 cells were generated using a CRISPR/Cas9 system, and three TET knockout cells contained mutations around targeting protospacer adjacent motif (PAM) sequences (Supplementary Figure S5A). A significant reduction in 5hmC levels was confirmed in NCI-H520 cells with the TET knockout (Figure 5B), along with an increase in DNA methylation at the 3’-UTR of the *ARL4C* DNA (Figure 5C). ARL4C mRNA and protein levels (Figure 5D), and cell migration activity (Figure 5E) were consistently reduced in TET knockout NCI-H520 cells. ARL4C expression in TET-knockout NCI-H520 cells #3 rescued the TET-knockout phenotypes (Figure 5F and 5G). In addition, five more TET-knockdown NCI-H520 cells were generated. In these clones 5hmC levels in genomic DNA were reduced, and DNA methylation at the 3’-UTR of the *ARL4C* gene was increased with a decrease in *ARL4C* mRNA levels (Supplementary Figure S5B-S5D). Therefore, the TET proteins are expected to be involved in ARL4C expression and function in lung SCC cells *in vitro*.

In addition, to examine whether the alternation in DNA methylation status at the 3’-UTR of the *ARL4C* gene affects transcription, chromatin immunoprecipitation assay using the antibody for trimethylated H3K4 (H3K4me3), which is preferentially detected in transcriptional active genes, was performed. In control NCI-H520 cells H3K4me3 was observed in the 3’-UTR methylation site, but in TET-knockout cells the level of H3K4me3 was greatly reduced, suggesting that DNA methylation status affects histone modification at the 3’-UTR of the *ARL4C* gene (Figure 5H). The putative binding site (+3605 to +3612) of cAMP-responsive element-binding protein (CREB) transcription factor was found around the 3’-UTR methylation sites using ECR Browser software (http://ecrbrowser.dcode.org). However, the interaction of CREB with the 3’-UTR of the *ARL4C* gene was not detected (Figure 5H). Therefore, we do not know at present whether the 3’-UTR methylation variation affects the interaction of transcriptional factors with the 3’-UTR of the *ARL4C* gene, but the 3’-UTR methylation could suppress *ARL4C* gene transcription.

### ARL4C is hypomethylated in the 3’-UTR in human lung SCC cases

**Figure 6:**
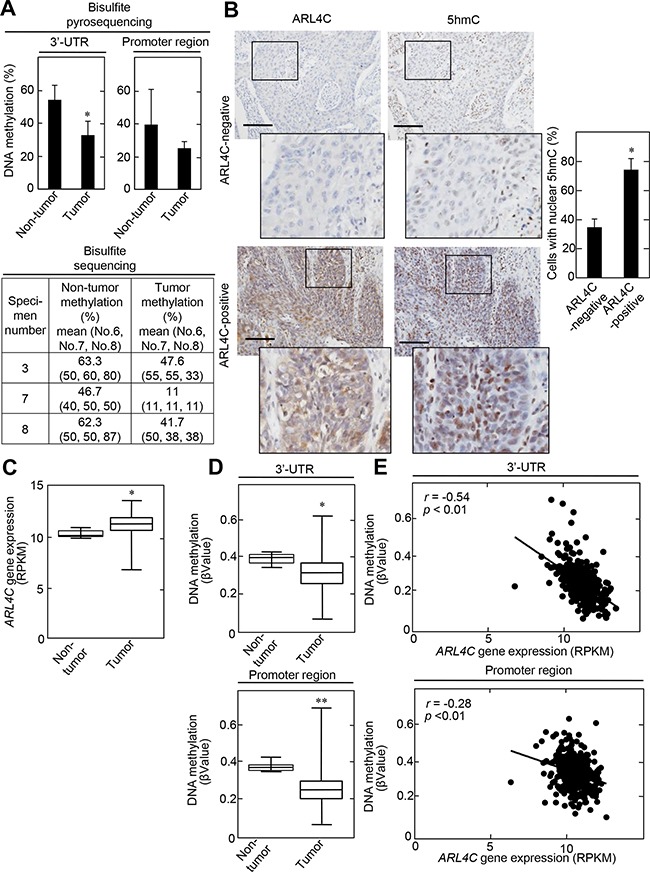
ARL4C methylation in lung SCCs **A.** Genomic DNA from 15 lung SCC tumor lesions, as well as from corresponding 10 non-tumor regions, were subjected to bisulfite pyrosequencing of the *ARL4C* 3’-UTR and promoter region. DNA methylation levels of the 3’-UTR indicate means of CpG No. 6 to No. 8 (upper panels). Genomic DNA from 3 lung SCC tumor lesions (specimen number 3, 7, and 8; see Supplementary Table S2), as well as from corresponding non-tumor regions, were subjected to bisulfite sequencing of the *ARL4C* 3’-UTR. Bisulfite sequencing was performed for CpG No. 6, No.7 and CpG No. 8 using different primers, because of technical difficulty in performing bisulfite sequencing for CpG No. 6 to No. 8 in one amplicon (lower panel). **B.** Lung SCC specimens were stained with anti-ARL4C or anti-5hmC antibodies and hematoxylin. Black boxes show enlarged images. Cells with nuclear 5hmC are expressed as the percentage of 5hmC-positive cells compared with total hematoxylin-stained cells in ARL4C -negative or -positive lesions (n = 7,046). Scale bars, 200 μm. **C.** *ARL4C* gene expression in 379 lung SCC cases, which were obtained from TCGA, was analyzed by using Illumina HiSeq and was expressed as RPKM (reads per kilobase of exon per million mapped). **D.** Illumina Infinium Human DNA Methylation 450 platforms were used for the methylation analysis of *ARL4C* DNA in the 3’-UTR or promoter region, and methylation level was expressed as βValue. **E.** Correlation between ARL4C DNA methylation (βValue, Y-axis) and gene expression (RPKM, X-axis) of *ARL4C* DNA in the 3’-UTR or promoter region was examined by Pearson's correlation analysis. ^*^, *P* < 0.01, ^**^, *P* < 0.05.

To elucidate whether ARL4C expression correlates to DNA methylation levels *in vivo*, genomic DNA was prepared from human lung SCC specimens with middle and high levels of ARL4C expression (see Supplementary Figure S1A), as well as from corresponding non-tumor regions, and then the methylation status of *ARL4C* DNA was also examined. Bisulfite pyrosequencing data showed that *ARL4C* DNA methylation levels are significantly reduced in the 3’-UTR in tumor lesions compared to non-tumor regions (Figure 6A and Supplementary Table S2). The methylation levels of the *ARL4C* promoter region (CpG islands) from tumor lesion tissue appeared to be reduced compared to non-tumor regions, although the difference was not significant. Bisulfite sequencing data indicated that methylation status of all of CpG No. 6, CpG No. 7, and CpG No. 8 was decreased in tumor lesions, suggesting that they were coordinately regulated (Figure 6A and Supplementary Figure S6A). Immunohistochemical analysis showed that 5hmC was observed in approximately 35% and 74% of the nuclei of ARL4C-negative and ARL4C-positive tumor lesion cells, respectively (Figure 6B). These results confirmed that ARL4C is expressed in tumor lesion cells where DNA methylation levels of the *ARL4C* gene were low.

The relationships between *ARL4C* gene expression and *ARL4C* DNA methylation in the 3’-UTR was further analyzed using lung SCC cohorts from The Cancer Genome Atlas (TCGA) dataset. Both parameters were available for 371 tumor lesions and 8 non-tumor regions in lung SCC cases. In the tumor lesions, *ARL4C* mRNA was elevated (Figure 6C) and DNA methylation status, in both the promoter region and the 3’-UTR, was decreased compared to those regions in non-tumor regions (Figure 6D). In addition, a significant inverse correlation between the level of *ARL4C* DNA methylation and gene expression was observed in the 3’-UTR more dominantly than the promoter region (Figure 6E) and especially in cg24441922 sites of the 3’-UTR (Supplementary Figure S6B), indicating that *ARL4C* DNA hypomethylation of the 3’-UTR might be essential for induction of *ARL4C* gene expression. These data-mining results are consistent with those obtained from our laboratory experiments.

The relationship between *ARL4C* mRNA expression and *ARL4C* DNA methylation in the 3’-UTR or promoter region was also examined using lung adenocarcinoma cohorts from TCGA dataset. Both parameters were available for 450 tumor lesions and 21 non-tumor regions in lung adenocarcinoma cases. In the tumor lesions, *ARL4C* mRNA was elevated (Supplementary Figure S7A) and this data is consistent with our previous immunohistochemical data [8]. DNA methylation status in the 3’-UTR was decreased in tumor lesions compared to that in non-tumor regions (Supplementary Figure S7B). In addition, a significant inverse correlation between the level of *ARL4C* DNA methylation and *ARL4C* mRNA expression was observed in the 3’-UTR more dominantly than the promoter region (Supplementary Figure S7C). Taken together, these results suggest that *ARL4C* DNA hypomethylation of the 3’-UTR might be involved in the induction of ARL4C expression in lung adenocarcinomas.

In addition, *TET* gene expression and its correlation with *ARL4C* gene expression or methylation status in lung SCC were examined using TCGA dataset. Although *TET2* mRNA expression positively correlated to *ARL4C* mRNA expression and the expression of *TET*s mRNA inversely correlated to *ARL4C* DNA hypomethylation both in the 3’-UTR and promoter region, *TET2* mRNA expression in tumor lesions was similar to non-tumor regions (Supplementary Figure S8A-S8C). These results suggest that at least TET2 functions to induce *ARL4C* mRNA expression through DNA hypomethylation, but whether elevated *TET2* mRNA expression in tumor lesions is required for inducing *ARL4C* mRNA expression in lung SCC is not clear.

## DISCUSSION

Our previous study demonstrated that ARL4C overexpression is detected in colon cancer and lung adenocarcinoma where genetic alterations in *APC*, *β-catenin*, and *Ras* are frequently observed [8]. In the present study, we found that ARL4C is also expressed in 70 ~ 80% of lung and tongue SCCs. It is noteworthy that ARL4C was clearly detected in the invasion front of tongue tumor lesions. Since ARL4C regulates cellular migration and invasion through the activation of Rac and the inactivation of Rho [6, 8], its overexpression in the peripheral areas of tumor lesions could promote invasiveness of cancer cells. Knockdown and knockout of ARL4C suppressed migration and proliferation of NCI-H520 and SAS cells. Since the activation of Wnt/β-catenin and EGF/Ras-MAPK signaling is not always clear in lung and tongue SCCs compared with colon cancers and lung adenocarcinomas, ARL4C might function as an oncogene in a certain types of cancers in which growth factor signaling is not constitutively activated.

During the lineage-specific differentiation of thymocyte progenitors from hematopoietic progenitors, ARL4C was found to be upregulated and its 3’-UTR was hypomethylated [24], indicating that ARL4C expression is regulated by DNA methylation in the T-DMRs. NCI-H520 cells highly express ARL4C with DNA hypomethylation in the 3’-UTR. Similarly, genomic DNA from tumor lesions of lung SCC patients also showed low levels of DNA methylation in the 3’-UTR compared to non-tumor regions of the same patient. In addition, lung SCC patients obtained from the TCGA dataset showed that *ARL4C* mRNA is highly expressed when the DNA is hypomethylated in the 3’-UTR and promoter region. A significant inverse correlation between the level of *ARL4C* DNA methylation and gene expression was observed more dominantly in the 3’-UTR compared with that in the promoter region. Lung adenocarcinoma patients from the TCGA dataset also showed high expression of *ARL4C* mRNA in tumor lesions with DNA hypomethylation in the 3’-UTR and inverse correlation between the levels of *ARL4C* DNA methylation and *ARL4C* mRNA expression. These results suggest that ARL4C overexpression is involved in tumorigenesis through *ARL4C* DNA hypomethylation in the 3’-UTR in lung cancer, such as SCC and adenocarcinoma.

Importantly, CpG No.6, CpG No.7, and CpG No.8 in the 3’-UTR were hypomethylated in cancer cells and the corresponding regions were also hypomethylated in T cell progenitors during lymphogenesis [24]. Thus, it is likely that the location of different methylation sites in cancer overlaps with that of T-DMRs, consistent with the previous observation [22]. These results support that ARL4C expression is regulated, not only by growth factor signaling, but also by alteration of DNA methylation in cancers. In addition, it is intriguing to speculate that tissue differentiation and cancer are affected by similar epigenetic mechanisms.

We found that Ets constitutively binds to the 3’-UTR (+3060 to +3067) of the mouse *Arl4c* gene, which corresponds to the 3’-UTR (+2852 to +2859) of the human *ARL4C* gene, and that a combination of Wnt3a and EGF induces formation of a complex between Ets, β-catenin, and Tcf4, enhancing *Arl4c* mRNA expression [6]. Since we showed that the 3’-UTR (+3622 to +3744) of the human *ARL4C* gene is demethylated by TET proteins, the 3’-UTR might be an essential site for the regulation of ARL4C expression by both growth factor signaling and epigenetic modification. Although we do not know which transcription factor(s) are controlled by methylation of CpG sites in the 3’-UTR, transcription of the *ARL4C* gene could be upregulated by hypomethylation of its CpG sites, because a ChIP assay revealed that the level of H3K4me3 is decreased in the 3’-UTR of TET-knockout NCI-H520 cells. Genome-wide methylation analysis revealed that 6% of T-DMRs are in islands, 76% of T-DMRs are located within 2 kb of islands (CpG island shores), and 18% of T-DMRs are located greater than 2 kb from the islands [22]. The 3’-UTR that is important for ARL4C expression would be categorized in the third group.

TET proteins convert 5mC to 5hmC, and they are known to function in various developmental stages [26, 27]. Recently, TET proteins were also reported to be involved in tumorigenesis through DNA demethylation by regulating tumor suppressor genes or oncogenes [28–30]. For example, *TET2* gene mutation and its catalytic inactivation are closely related to acute myeloid leukemia [31–33]. Leukemia-associated TET2 mutations cause loss of function with respect to TET2-mediated hydroxymethylation, leading to increased cytosine methylation in patients with TET2 mutations [34]. However, it is unclear how TET proteins bind to a specific locus in the genome and how they regulate different tumor-related genes.

Direct correlation between ARL4C expression and TET expression was not examined, because antibodies for the immunohistochemical study of TET proteins in human lung SCC specimens were not available. However, immunohistochemical analysis showed that ARL4C was highly expressed in tumor lesions where 5hmC levels were also high (Figure 6B). Therefore, ARL4C might be a target gene of TET proteins in tumorigenesis and the 3’-UTR of *ARL4C* DNA would be a specific locus for TET protein binding.

Knockdown of three TETs by siRNA, but not that of one or two TETs, reduced the expression of *ARL4C* mRNA. Tet1-deficient mouse embryonic stem cells (mESCs) maintained pluripotency and were largely normal [35], whereas knockout of both Tet1 and Tet2 or three Tets in mESCs showed severe abnormalities in differentiation [36, 37]. It was also reported that knockdown of Tet1 or Tet2 minimally affects pluripotency gene *Nanog* mRNA expression, but knockdown of both Tet1 and Tet2 reduces *Nanog* mRNA expression significantly in mESCs [38] and that TET1, TET2, and TET3 are required for the expression of the tumor suppressor gene *TCF21* mRNA in cancer cells [39]. Therefore, TET family members have the redundant functions for the expression of certain genes in embryogenesis and tumorigenesis. The *ARL4C* gene could be one of the examples. In addition, the TCGA dataset revealed that the function of TET2 might be important to induce *ARL4C* gene expression in lung SCC, but whether elevated TET2 expression in tumor lesions is required for the expression of ARL4C remains to be clarified.

In summary, we found that ARL4C is overexpressed in tumor lesions of lung and tongue SCC cancers with high frequencies and that ARL4C expression is regulated by DNA methylation at the 3’-UTR through TETs. Since we already demonstrated that ARL4C is overexpressed in lung adenocarcinoma and colon cancer through aberrant activation of growth factor signaling and that ARL4C depletion suppressed cancer cell proliferation and migration [8]. Taken together, these results suggest that ARL4C contributes to oncogenesis as a result of its overexpression by multiple mechanisms.

## MATERIALS AND METHODS

### Patients and cancer tissues

Lung squamous cell carcinoma (n = 62) and tongue squamous cell carcinoma (n = 57) tissue from patients who underwent surgery at Osaka University Hospital and Osaka University Dental Hospital, respectively, from January 2010 to March 2015, were examined in this study. The ages of the lung squamous cell carcinoma patients ranged from 28 to 85 years (median, 71 years) and tongue squamous cell carcinoma patients ranged from 22 to 81 years (median, 63 years). Tumors were staged according to the International Union Against Cancer (UICC) TNM staging system and the WHO (World Health Organization) Classification [40]. Resected specimens were macroscopically examined to determine the location and size of a tumor, and specimens for histology were fixed in 10% (v/v) formalin and processed for paraffin embedding. Specimens for examination were sectioned at 4 μm thickness and stained with hematoxylin and eosin or immunoperoxidase for independent evaluations by four pathologists (S.N., E.M., Y.U., and S.T). The protocol for this study was approved by the ethical review board of the Graduate School of Medicine, Osaka University, Japan (No. 13552) and the Ethical Committee of the Osaka University Dental Hospital, Osaka University, Japan (H26-E20).

### Immunohistochemical studies

Immunohistochemical studies were performed as previously described [8, 41, 42] with modification. Tissue sections for immunohistochemical staining were examined using a DakoReal™EnVision™ Detection System (Dako, Carpentaria, CA, USA) in accordance with the manufacturer's recommendations. Antigen retrieval for staining was done using a decloaking chamber (Biocare Medical, Walnut Creek, CA, USA). Endogenous peroxidase activity was blocked with 3% H_2_O_2_-methanol for 15 minutes, and the sections were then incubated with goat serum for 1 hour to block nonspecific antibody binding sites. Tissue specimens were incubated with rabbit anti-ARL4C (1:100) or rabbit anti-5hmC (1:500) antibody for 16 hours at 4°C, and binding was detected by subsequent incubation with goat anti-rabbit IgG-horseradish peroxidase (HRP) for 1 hour. Diaminobenzidine (DAB) (Dako) was used as a chromogen. The tissue sections were then counterstained with 0.1% (w/v) hematoxylin. Tumors in which the positively stained area covered > 5% were classified as ARL4C-positive or 5hmC-positive.

### Cells and antibodies

HeLaS3 uterine cancer, A549 lung adenocarcinoma, NCI-H520 lung SCC, and SAS tongue SCC cells were kindly provided from Dr. K. Matsumoto (Nagoya University, Aichi, Japan) in May 2002, Dr. Y. Shintani (Osaka University, Suita, Japan) in September 2013, and Dr. Y. Usami (Osaka University, Suita, Japan) in August 2013, respectively, and the cells were authenticated at the end of the experiments by species-specific polymerase chain reaction (PCR) in April 2014 (ICLAS Monitoring Center, Kanagawa, Japan) and used within 6 months after authentication. All cells were permanently stocked at liquid nitrogen when obtained. Lenti-X™ 293T (X293T) cells were purchased from Takara Bio Inc. (Shiga, Japan). HeLaS3, A549 and X293T cells were grown in Dulbecco's modified Eagle's medium (DMEM) supplemented with 10% fetal bovine serum (FBS). NCI-H520 and SAS cells were grown in RPMI-1640 supplemented with 10% FBS. HeLaS3 and NCI-H520 cells were treated with 50 μM 5-azacytidine (5-AzaCR, Wako Pure Chemical Industries, Osaka, Japan) or 50 μM Zebularine (Wako) for 5 days with the drug and medium replaced every 24 hours [43–46].

Anti-ARL4C antibody and anti-double strand (ds) DNA antibody were from Abcam (Cambridge, UK). The specificity of the anti-ARL4C antibody was confirmed by the reduction of ARL4C signal by ARL4C siRNA [8]. Anti-HSP90 antibody and anti-β-catenin antibody were from BD Biosciences (San Jose, CA, USA). Anti-phospho-p44/42 (ERK1/2) (Thr202/Tyr204) and anti-p44/42 (ERK1/2) antibodies were from Cell Signaling Technology (Beverly, MA, USA). β-Tubulin and anti-5hmC antibodies were from Sigma-Aldrich (Steinheim, Germany) and active motif (Carlsbad, CA, USA), respectively. Anti-GFP antibody was from Invitrogen (Carlsbad, CA, USA).

### The cancer genome atlas data (TCGA)

As previously reported [43], *ARL4C* gene expression and DNA methylation data for 379 lung SCC or 471 lung adenocarcinoma cases were obtained from the TCGA web site (http://tcga-data.nci.nih.gov/tcga/tcgaHome2.jsp). Illumina HiSeq was used for gene expression. Methylation was analyzed using the Illumina Infinium Human DNA Methylation 450 platform, and a βvalue > 0.2 was considered as methylation-positive. We used Probe ID on the Infinium HumanMethylation 450 array, cg24441922, cg05204104, and cg15016771 for the 3’-UTR or cg13539030, cg09453076, cg05308656, cg15235893 and cg09935994 for the promoter region of *ARL4C* DNA, respectively.

### Generation of *ARL4C* or *TET* knockout cells

The target sequence for human *ARL4C*, 5’-CTTCTCGGTGTTGAAGCCGA-3’, was designed with the help of the CRISPR Genome Engineering Resources (http://www.genome-engineering.org/crispr/), [47] and the oligonucleotides for TET1, TET2, and TET3 were previously described [48]. The plasmids expressing hCas9 and single-guide RNA (sgRNA) were prepared by ligating oligonucleotides into the BbsI site of pX330 (addgene#42230). The plasmid pX330 with sgRNA sequences targeting ARL4C, TET1, TET2, or TET3 and Blasticidin resistance was introduced into NCI-H520 or SAS cells using Lipofectamine LTX reagent (Invitrogen) according to manufacturer's instructions and the transfected cells were selected in medium containing 5 μg/mL Blasticidin S for two days [49]. Single colonies were picked, mechanically disaggregated, and replated into individual wells of 24-well plates. To generate TET family knockout cells, sgRNA sequences targeting TET1, TET2, and TET3 were transfected at the same time.

### Plasmid construction and infection using lentivirus harboring a cDNA

The pEGFPN3-ARL4C and ARL4C plasmids were constructed as previously described [6]. Lentiviral vectors were constructed by subcloning GFP, pEGFPN3-ARL4C, and ARL4C cDNAs into CSII-CMV-MCS-IRES2-Bsd, which was kindly provided by Dr. H. Miyoshi (RIKEN BioResource Center, Ibaraki, Japan)[50]. Standard recombinant DNA techniques were used to design a plasmid harboring ARL4C for rescue experiments. The nucleotides coding for the threonine, isoleucine, glycine resides at positions of 44, 45, 46, respectively, which correspond to the protospacer adjacent motif (PAM) sequences, were mutagenized without changing the amino acids. The vectors were then transfected along with the packaging vectors, pCAG-HIV-gp and pCMV-VSV-G-RSV-Rev, into X293T cells using the FuGENE HD transfection reagent (Roche Applied Science, Basel, Switzerland) to generate lentiviruses.

To generate NCI-H520 and SAS cells that stably expressed ARL4C-GFP or ARL4C for rescue experiments, parental cells (5 x 10^4^ cells/well in a 12-well plate) were treated with lentivirus and 10 μg/mL polybrene. The cells were then centrifuged at 1200 x *g* for 1 hour, and incubated for another 24 hours. The cells that demonstrated stable expression of ARL4C-GFP, or ARL4C for rescue experiments, were selected and maintained in culture medium containing 5 μg/mL Blasticidin S.

### DNA methylation analysis and dot blot analysis

Genomic DNA from lung squamous cell carcinoma specimens was extracted using WaxFree™ Paraffin Sample DNA Extraction Kit (TrimGen, Sparks, MD, USA). Bisulfite treatment on the extracted genomic DNA was performed as described previously [24, 43, 51]. The DNA methylation levels were measured using bisulfite pyrosequencing or bisulfite sequencing technology. Primer sequences and PCR conditions are shown in Supplementary Table S1.

Dot blot analysis was performed as previously described [30, 48] with modification. Genomic DNA was diluted to 50 ng/μL in 20 μL total volume. Five μL of 0.5 M NaOH was added to each sample and then the samples were incubated at 99°C for 5 minutes. Samples were put on ice and neutralized with 2.5 μL of 6.6 M Ammonium Acetate. 2.75 μL of each mixture was spotted onto a nitrocellulose membrane and allowed to air dry for 10 minutes. The membrane was baked for 2 hours at 80°C and blocked with 5 % milk for 2 hours at room temperature. Anti-5hmC antibody (1:10000) or anti-ds DNA antibody (1:1000) were incubated for 12 hours at 4°C, and subsequent incubation with goat anti-rabbit IgG-HRP or goat anti-mouse IgG-HRP, respectively, for 1 hour. ECL Western Blotting Detection Reagents (GE Healthcare, Little Chalfont, UK) were used for detection.

### Chromatin immunoprecipitation (ChIP) assay

ChIP assay was performed as described previously [6]. Control or TET knockout NCI-H520 cells (1×10^7^) were cross-linked with 1% formaldehyde for 10 min at room temperature. The cell pellets were lysed with sodium dodecyl sulfate (SDS) lysis buffer (50 mM Tris/HCl [pH 8.0], 10 mM EDTA, and 0.5% SDS) and sonicated to shear DNA to a size range between 200 and 1000 bp. Sheared chromatin samples were diluted in ChIP dilution buffer (16.7 mM Tris/HCl [pH 8.0], 167 mM NaCl, 1.2 mM EDTA, and 1.1% Triton X-100) supplemented with protease inhibitors, and precleared with salmon sperm DNA/protein A-agarose (Millipore, Billerica, MA, USA) and incubated for 12 h at 4°C with 5 mg of histone H3 (tri methyl K4) (Millipore) and anti-CREB (Abcam) antibodies, or negative control IgG (Diagenode*,* Liége*,* Belgium). Immunocomplexes were absorbed with salmon sperm DNA/protein A-agarose beads, and washed once with high salt buffer (20 mM Tris/HCl [pH 8.1], 500 mM NaCl, 0.1% SDS, 1% TritonX-100, and 2 mM EDTA), once with LiCl buffer (10 mM Tris/HCl [pH 8.1], 0.25 M LiCl, 1 mM EDTA, 1% deoxycholic acid, and 1% Nonidet P-40), and three times with TE buffer (10 mM Tris/HCl (pH 8.1), and 1 mM EDTA). Immune complexes extracted in elution buffer (1% SDS and 100 mM NaHCO_3_ ) were incubated for 4 h at 65°C to revert DNA-protein cross-links. Then the DNA was extracted by incubation in proteinase K (final concentration of 50 mg/ml) buffer for 1 h at 45 °C. The purified DNA was used in PCR to assess the presence of target sequences. Forward and reverse primers were as follows: 5’-GGAGGCCAACTTCCCCTAT-3’ and 5’- TGCAGTAAAGTAAAGCCCTGTG -3.

### Knockdown of protein expression by siRNA

The effects of protein knockdown by siRNA were analyzed as previously described [6, 8]. The following target sequences were used. Randomized control, 5’-CAGTCGCGTTTGCGACTGG-3’; human ARL4C #1, 5’-GGCTGTGAAGCTGAGTAAT-3’; human ARL4C #2, 5’-GAGTGCGTCAAGAAAGAAT-3’; human β-catenin, 5’- GTCCTGTATGAGTGGGAAC-3’; human TET1, 5’-GCAGCTAATGAAGGTCCAGAAC-3’; human TET 2, 5’-GCTACAGGAAACATGAATA-3’; and human TET3, 5’-GCCTCCTTCTCCTTTGGTT-3’. NCI-H520 and SAS cells were transfected with a mixture of siRNAs (20 nM each) against genes of interest using RNAiMAX (Invitrogen). The transfected cells were then used for experiments conducted at 36-48 hours post-transfection.

### Digital PCR

Digital PCR was performed by using a QuantStudio™ 3D digital PCR system (Life Technologies, Carlsbad, CA) to detect human TET1, TET2, TET3, DNMT1, DNMT3a, DNMT3b, and GAPDH according to the manufacturer's instructions. We used TaqMan^®^ Assay ID probe Hs00286756_m1, Hs00325999_m1, Hs00379125_m1, Hs00154749_m1, Hs01027166_m1, Hs00171876_m1, Hs99999905_m1, respectively. For Digital PCR, 80 ng for TET1, TET2, TET3, DNMT1, DNMT3a and DNMT3b or 1 ng for GAPDH of cDNA were applied to following protocol: 96 °C for 10 min, 39 cycles at 60 °C for 2 min, 98 °C for 30 s, and 60 °C for 2 min. The data was analyzed with the QuantStudio™ 3D AnalysisSuite™ v3.0 for quantification of each gene's copy numbers.

### Quantitative RT-PCR

Quantitative RT-PCR was performed as described previously [52]. Forward and reverse primers were as follows: human ARL4C, 5’-GTGCTCTACC GGCTCAAGTT-3’ and

5’-ACCGAGTCCACCACG TAGAT-3’; human TET1, 5’-CACACCAGCTCCA CTGAAGA-3’ and 5’-CTCCATCACAGGAGCAG ACA-3’; human TET2, 5’-CCCACTTACCTGC GTTTCAT-3’ and 5’-ACTGTGACCTTTCCCCACTG-3’; human TET3, 5’- AAAGAAACGGAAACGGTGTG-3’ and 5’-GCTGAGCTCTGAGCCTGTCT-3’; human DNMT1, 5’- GAACGGTGCTCATGCTTACA-3’ and 5’- TGTAATCCTGGGGCTAGGTG-3’; human DNMT3a, 5’-AGCCCAAGGTCAAGGAGATT-3’ and 5’-CAG CAGATGGTGCAGTAGGA-3’; human DNMT3b, 5’-TTGAATATGAAGCCCCCAAG-3’ and5’-GGTTCC AACAGCAATGGACT-3’; human GAPDH,5’-AGCCCA GAACATCATCCCTG-3’ and 5’- CACCACCTTCTTG ATGTCATC-3’.

### Cell migration assay

Transwell assay using a modified Boyden chamber (tissue culture treated, 6.5 mm in diameter, 10 μm thick, 8 μm pores; Transwell, Costar, Cambridge, MA, USA) were conducted to measure cell migration activity as previously described [53–55]. The lower surface of the filter was coated with 10 μg/mL type I collagen for 2 hours. Then, NCI-H520 and SAS cells (2.5 x 10^4^ cells in 100 μL) suspended in serum-free DMEM containing 0.1% (w/v) bovine serum albumin (BSA) were applied to the upper chamber. The cells were incubated for 4 to 8 hours at 37°C, and cells that had migrated to the lower side of the upper chamber were fixed with PBS containing 4% (w/v) paraformaldehyde (PFA) and counted.

### Statistical analysis

Statistical analyses were performed using JMP software (SAS Institute. Inc., Cary NC, USA). Differences in results were tested for statistical significance using the Fisher exact test for Table 1 and Student's *t*-test for other experiments. Pearson's correlation was used to examine the correlation between ARL4C DNA methylation and gene expression. *P* values of < 0.01 or < 0.05 were considered statistically significant.

### Additional assays

3D Matrigel culture of SAS cells was performed as previously described [6, 8]. Cell proliferation were performed as described [53–55]. Western blotting data are representative of at least three independent experiments.

## SUPPLEMENTARY MATERIALS FIGURES AND TABLES


