# Supplementary material for: Epigenetic upregulation of ARL4C, due to DNA hypomethylation in the 3'-untranslated region, promotes tumorigenesis of lung squamous cell carcinoma
Source: Oncotarget. 2016 Nov 7;7(49):81571–87. doi: 10.18632/oncotarget.13147 (PMC5348413; doi:10.18632/oncotarget.13147)
Supplement: Supplementary file 1 [file oncotarget-07-81571-s001.pdf]

Epigenetic upregulation of ARL4C, due to DNA hypomethylation in the 3′-untranslated region, promotes tumorigenesis of lung squamous cell carcinoma

SUPPLEMENTARY FIGURES AND TABLES

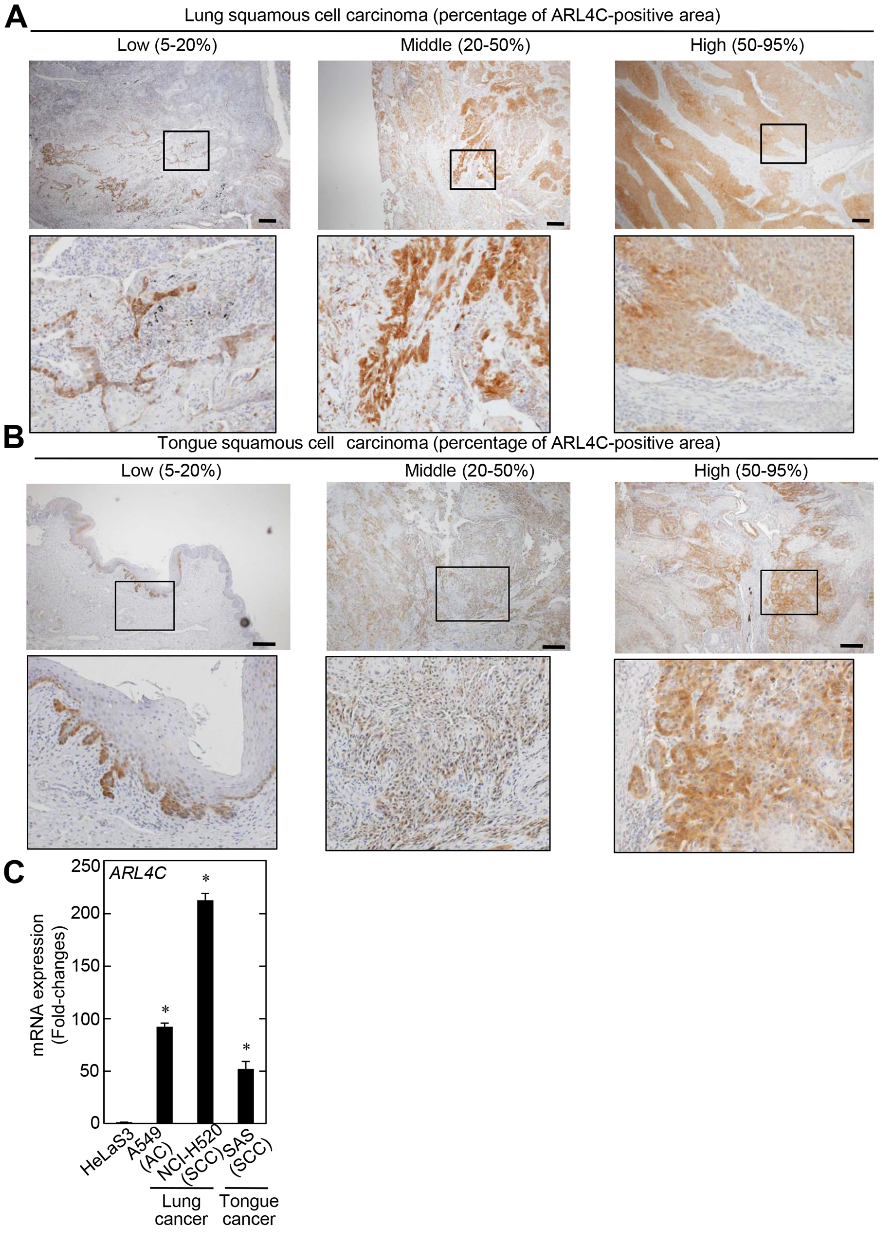

Supplementary Figure S1: ARL4C expression in lung and tongue squamous cell carcinomas. (A and B) Lung **A.** and tongue **B.** squamous cell carcinoma specimens were stained with anti-ARL4C antibody and hematoxylin. Black boxes show enlarged images. Areas staining positive for ARL4C were classified as follows. Low, 5-20%; middle, 20-50%; high, 50-95%. Scale bars, 200 µm. **C.** *ARL4C* mRNA levels in HeLaS3, A549, NCI-H520, and SAS cells were measured by quantitative RT-PCR. Relative levels of *ARL4C* mRNA expression were normalized to *GAPDH* and expressed as fold-changes compared with expression in HeLaS3 cells. AC, adenocarcinoma; SCC, squamous cell carcinoma.

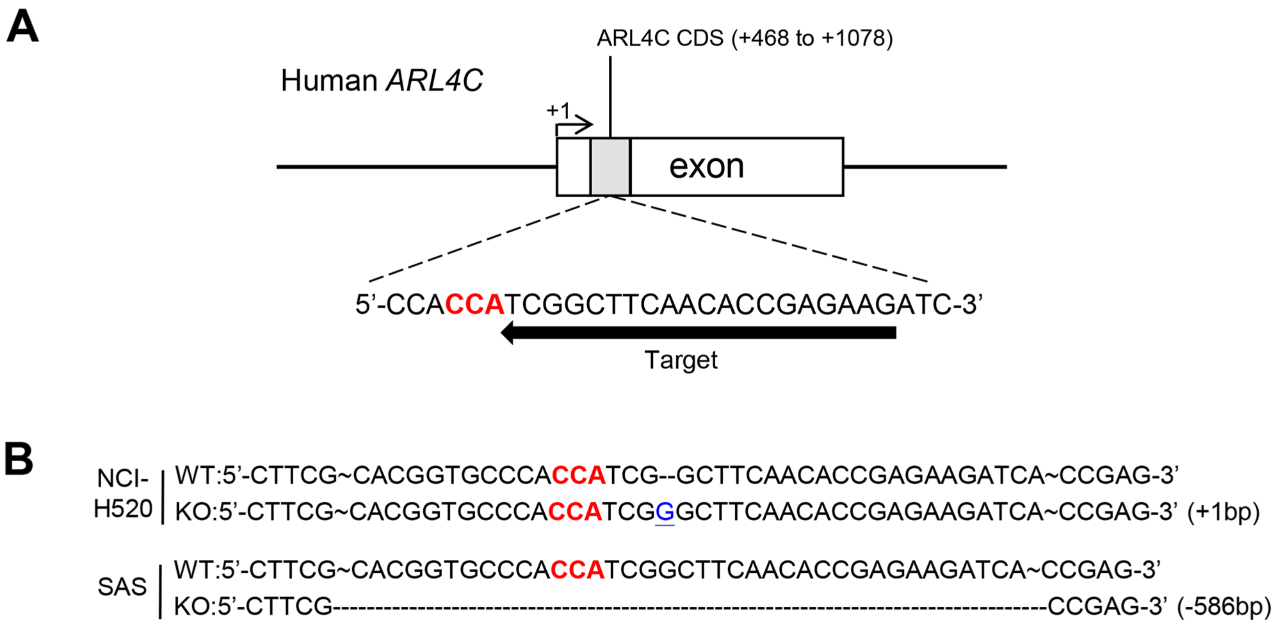

Supplementary Figure S2: Generation of ARL4C knockout cells. **A.** Schematic drawing of the targeting site of the single guide RNA at exon of human *ARL4C* gene. **B.** Sequences of ARL4C with PAM sequences labeled in red in knockout NCI-H520 or SAS cells are shown. Blue letters indicate mutated nucleotides.

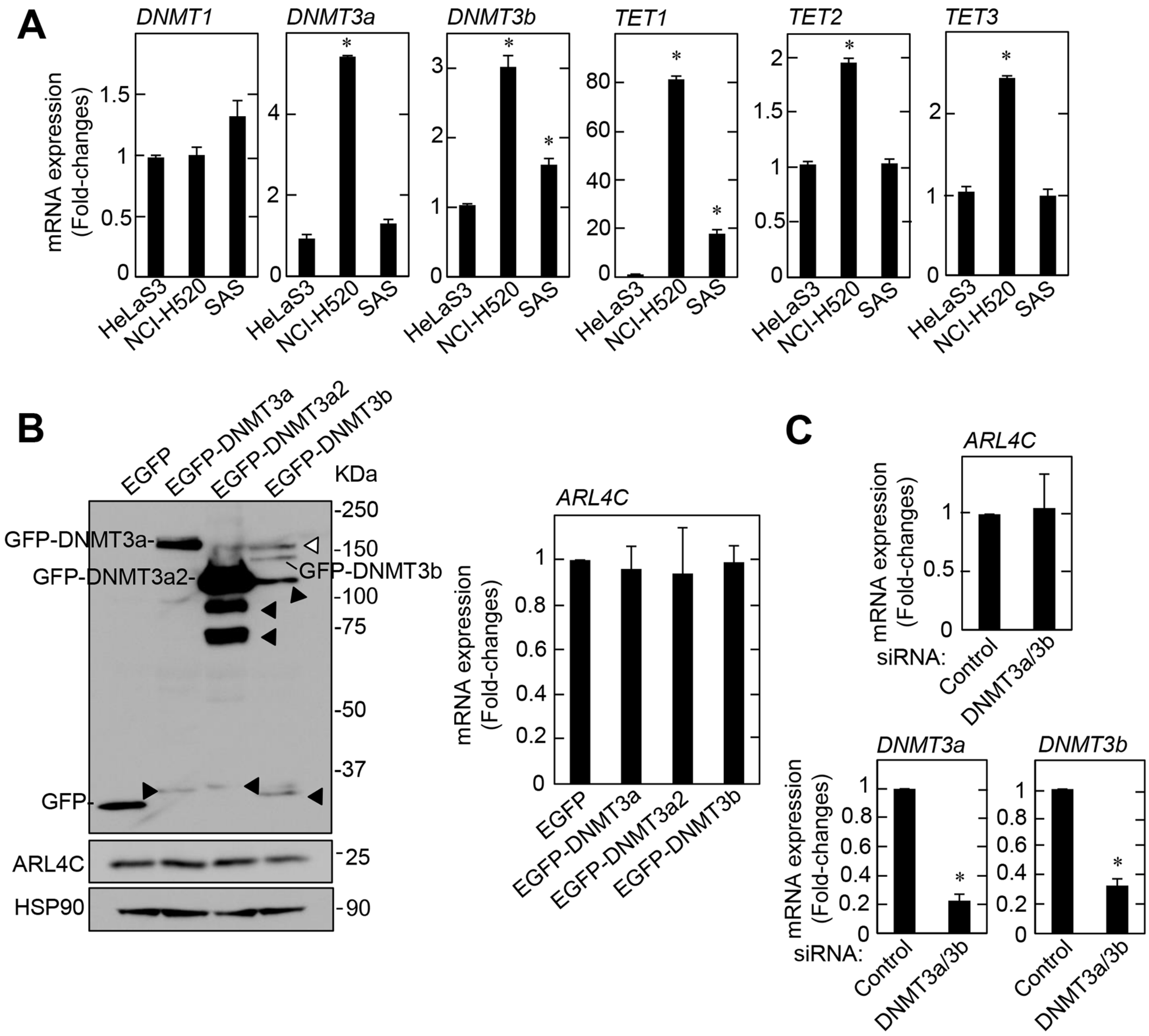

Supplementary Figure S3: Effects of overexpression or knockdown of DNMT on ARL4C expression. A. *DNMT1, DNMT3a, DNMT3b, TET1, TET2,* and *TET3* mRNA levels in HeLaS3, NCI-H520, and SAS cells were measured by quantitative RT-PCR. Relative levels of their mRNA expression were normalized to *GAPDH* and expressed as fold-changes compared with expression in HeLaS3 cells. **B.** NCI-H520 cells were transfected with EGFP, EGFP-DNMT3a, EGFP-DNMT3a2, and EGFP-DNMT3b for 48 hours and *ARL4C* mRNA levels were measured by quantitative RT-PCR. Relative *ARL4C* mRNA levels were normalized to *GAPDH* and expressed as fold-changes compared with levels in EGFP transfected cells. Cell lysates were probed with anti-GFP, anti-ARL4C, and anti-HSP90 antibodies. The arrowheads indicate degradation products of exogenously expressed EGFP-DNMTs and open triangle indicates non-specific bands. **C.** HeLaS3 cells were transfected with control siRNA or combinations of siRNA for DNMT3a and DNMT3b, and *ARL4C, DNMT3a* and *DNMT3b* mRNA levels were measured by quantitative RT-PCR. Relative levels of their mRNA expression were normalized to *GAPDH* and expressed as fold-changes compared with expression in control siRNA transfected cells. *, P < 0.01.

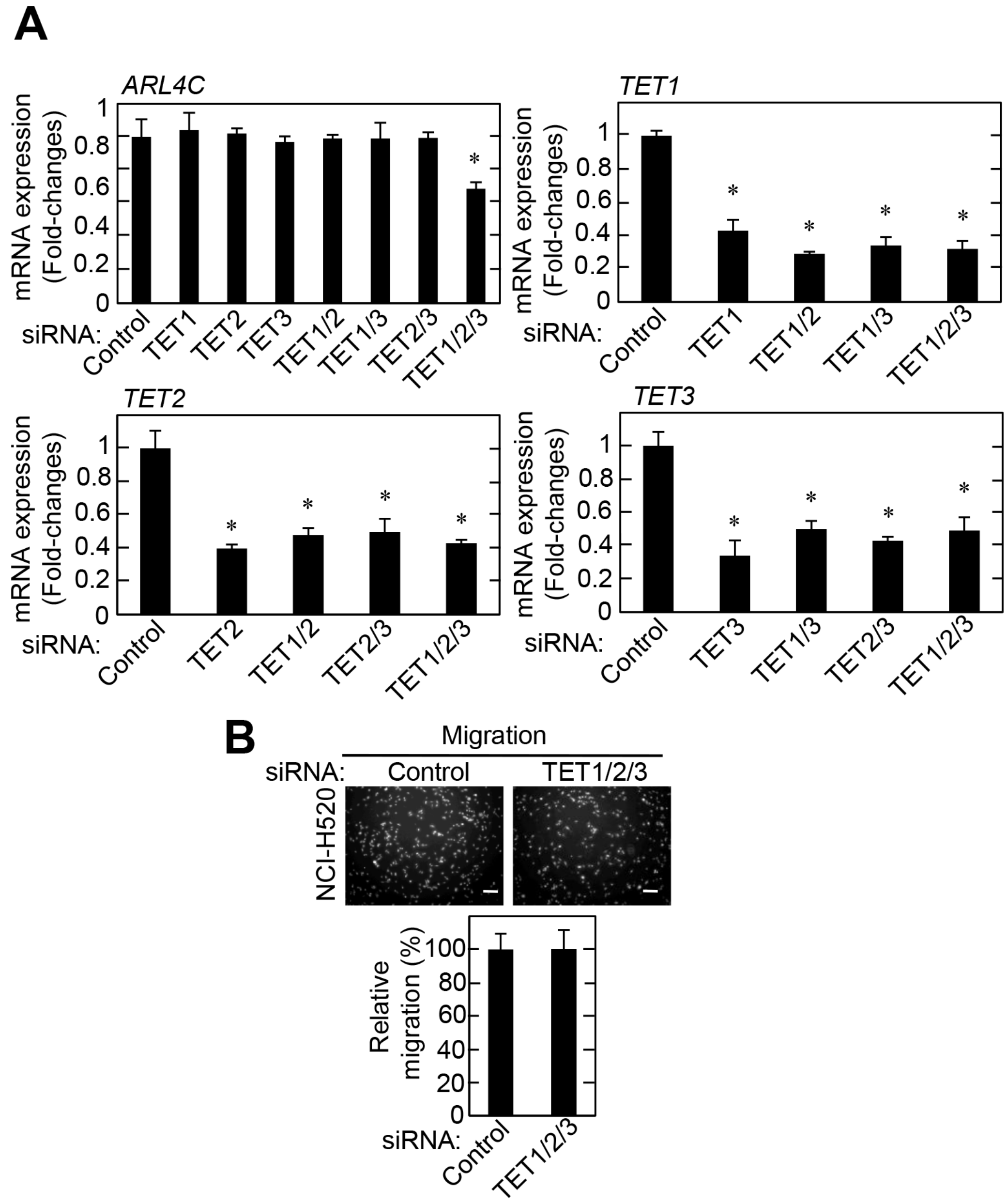

Supplementary Figure S4: Effects of knockdown of TET on ARL4C expression. A. NCI-H520 cells were transfected with control siRNA or various combinations of siRNA for TET1, TET2, and TET3, and *ARL4C, TET1, TET2*, or *TET3* mRNA levels were measured by quantitative RT-PCR. Relative levels of their mRNA expression were normalized to *GAPDH* and expressed as fold-changes compared with expression in control siRNA transfected cells. **B.** NCI-H520 cells were transfected with control siRNA or all siRNAs for TET1, TET2, and TET3 and placed in Transwell chamber for the migration assays. Migration activities are expressed as the percentage of control cells. *, $P < 0.01$. Scale bars in B are 200 μm.

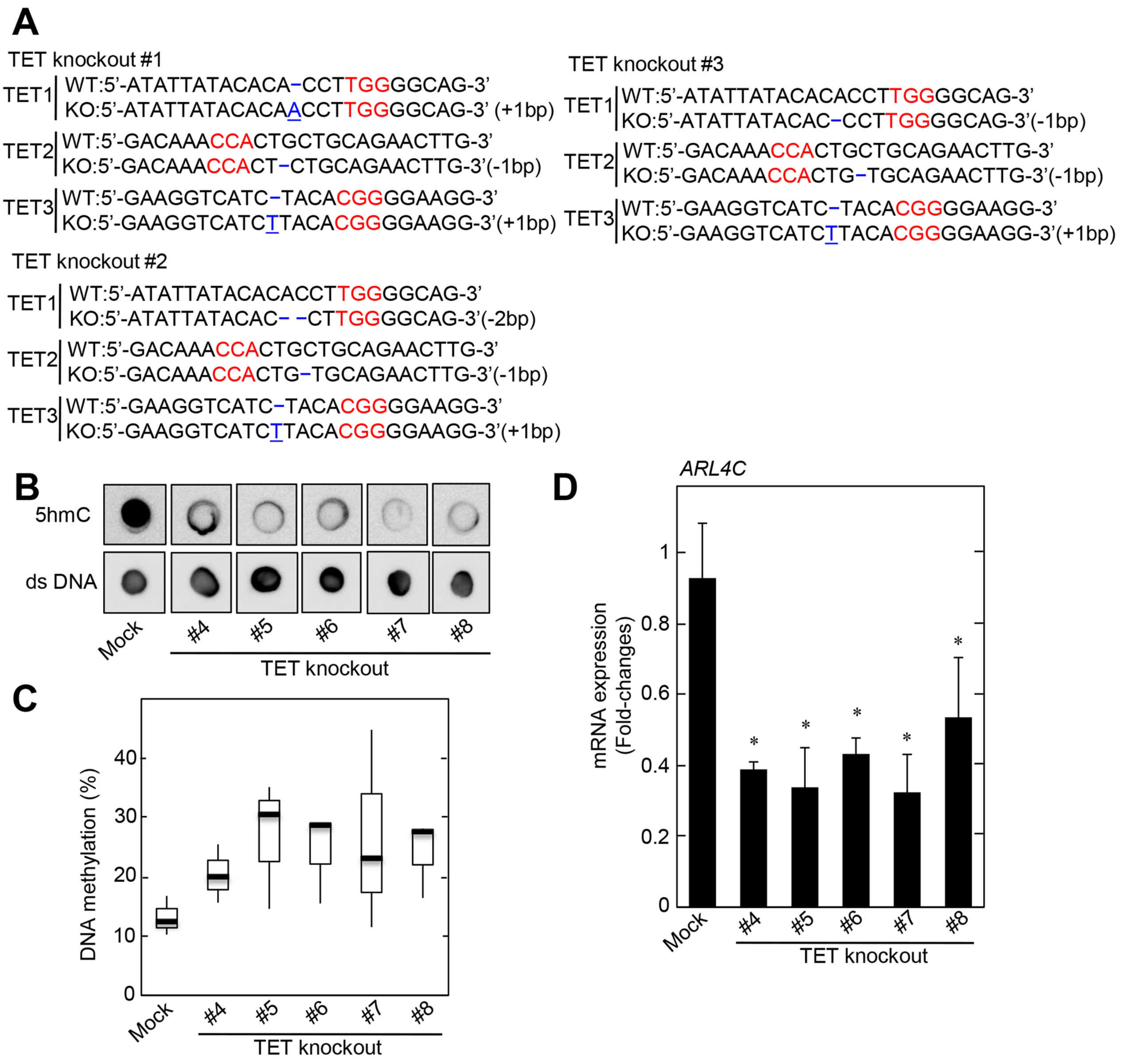

Supplementary Figure S5: Effects of knockout of TETs on ARL4C expression. A. TET family knockout NCI-H520 cells were generated and sequences of the TET family knockout NCI-H520 cell clones with protospacer adjacent motif (PAM) sequences labeled in red are shown. Blue letters indicate mutated nucleotides. **B.** Analysis of 5hmC levels in genomic DNA isolated from TET family knockout NCI-H520 cell clones was performed by dot blot assay using an anti-5hmC antibody. Anti-double strand (ds) DNA antibody was probed as a control. **C.** DNA methylation status of the *ARL4C* 3'-UTR in TET family knockout NCI-H520 cells was examined by bisulfite pyrosequencing. **D.** *ARL4C* mRNA levels in TET family knockout NCI-H520 cells were measured by quantitative RT-PCR and relative levels of *ARL4C* mRNA expression were normalized to *GAPDH* and expressed as fold-changes compared with control cells. Results are shown as means ± s.d. of three independent experiments. *, $P < 0.01$.

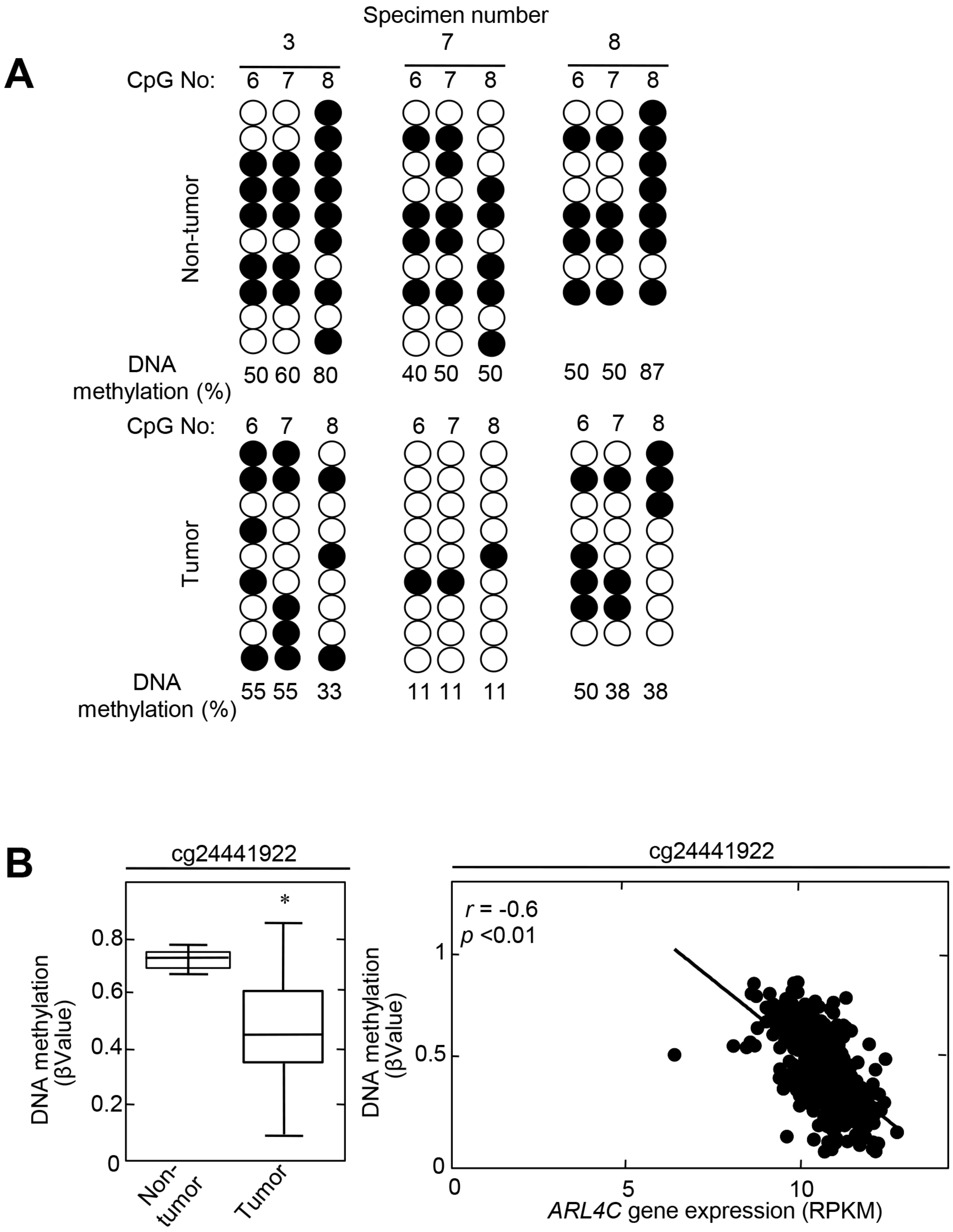

Supplementary Figure S6: *ARL4C* DNA methylation in lung SCCs. A. Genomic DNA from 3 lung SCC tumor lesions (specimen number 3, 7, and 8; see Supplemental Table 2), as well as from corresponding non-tumor regions, were subjected to bisulfite sequencing of the ARL4C 3'-UTR. DNA methylation levels of each CpG No. 6, 7 and 8 are shown. Empty dots indicate unmethylated CpGs; black dots indicate methylated CpGs. **B.** Illumina Infinium Human DNA Methylation 450 platforms were used for methylation analysis of ARL4C DNA in cg24441922 sites of the 3'-UTR, and methylation level was expressed as β Value (left graph). Correlation between *ARL4C* DNA methylation (β Value, Y-axis) and gene expression (RPKM, X-axis) of *ARL4C* DNA in cg24441922 sites of the 3'-UTR was examined by Pearson's correlation analysis (right graph).

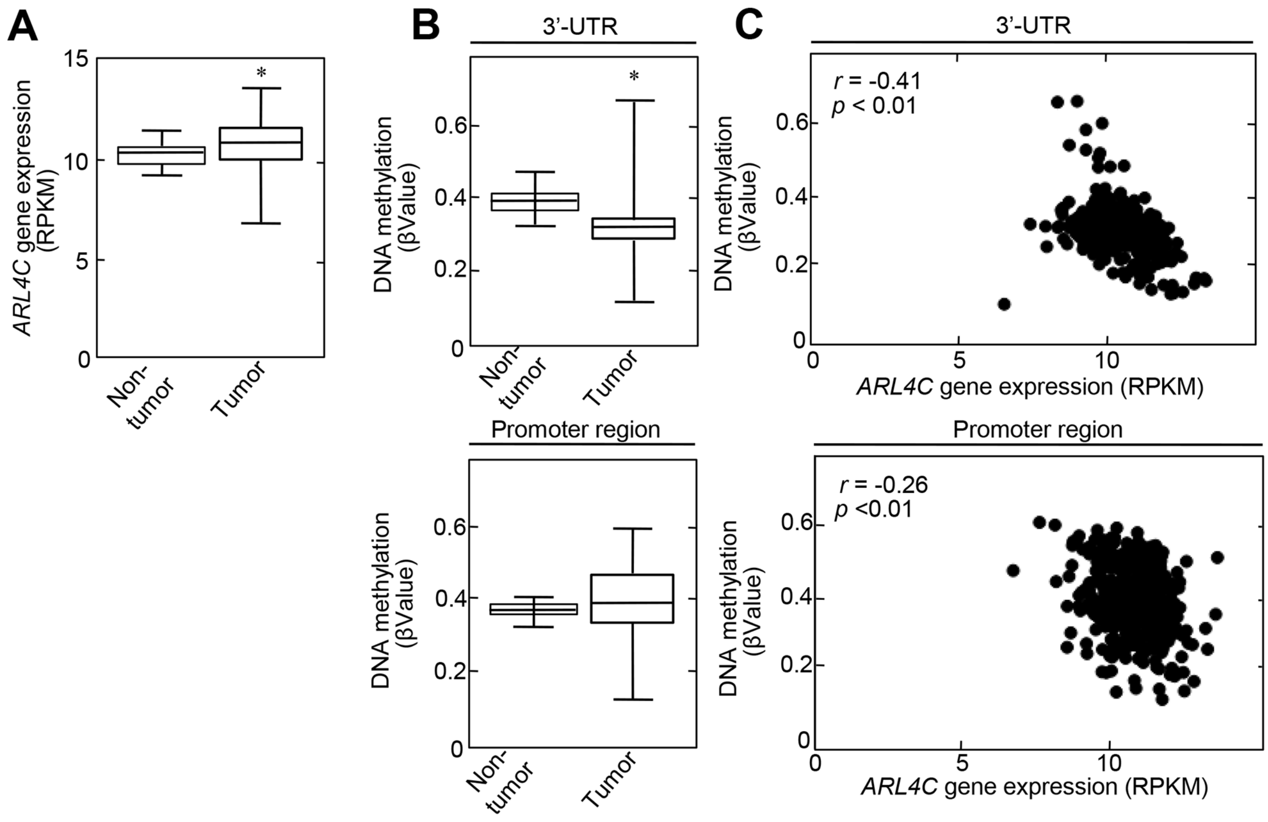

Supplementary Figure S7: *ARL4C* DNA methylation in lung adenocarcinomas. A. *ARL4C* gene expression in 471 lung adenocarcinoma cases, which were obtained from TCGA, was analyzed by using Illumina HiSeq and was expressed as RPKM. **B.** Illumina Infinium Human DNA Methylation 450 platforms were used for the methylation analysis of *ARL4C* DNA in the 3'-UTR or promoter region, and methylation level was expressed as β Value. **C.** Correlation between *ARL4C* DNA methylation (β Value, Y-axis) and gene expression (RPKM, X-axis) of *ARL4C* DNA in the 3'-UTR or promoter region was examined by Pearson's correlation analysis. *, P < 0.01.

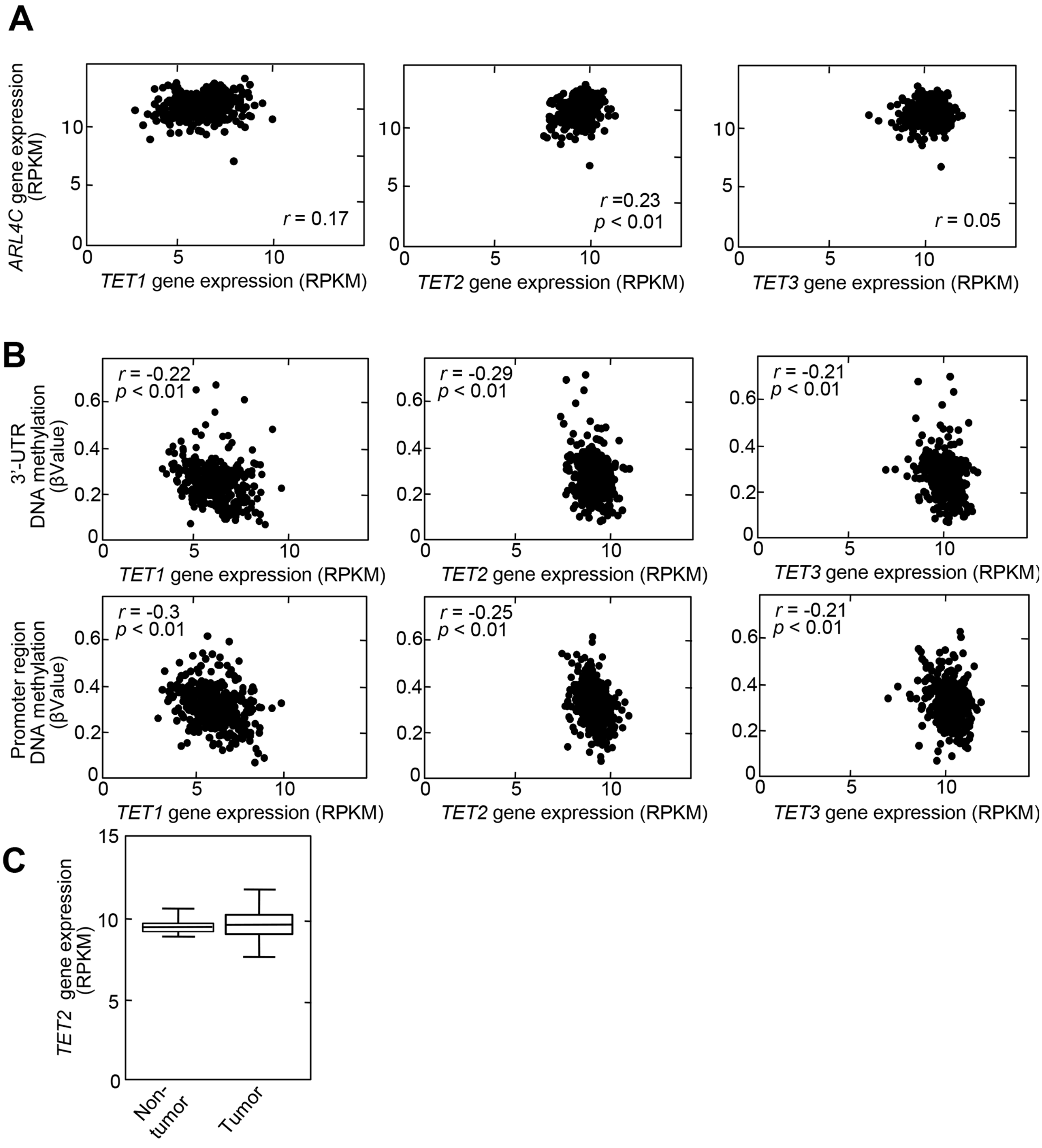

Supplementary Figure S8: *TET* gene expression and *ARL4C* DNA methylation in lung SCCs. A. Correlation between *ARL4C* gene expression (RPKM, Y-axis) and *TET* gene expression (RPKM, X-axis) in 379 lung SCC cases, which were obtained from TCGA, was analyzed by using Illumina HiSeq and was examined by Pearson's correlation analysis. **B.** Correlation between *ARL4C* DNA methylation status (β Value, Y-axis) in the 3'-UTR or promoter region and *TET* gene expression (RPKM, X-axis) was analyzed by using Illumina Infinium Human DNA Methylation 450 platforms and was examined by Pearson's correlation.

Supplementary Table S1: Primer sequences for pyrosequencing methylation analysis

Gene	Forward primer	Reverse primer	Sequence primer	Annealing temperature
ARL4C (CpG No.6 and No.7)	GGGTGTAATTAA TTGTTAGTAATTG	GGGACACCGCTGATCGTTTAAA CCACTCCAAAATAATTTAATCT	TAATGTTTG ATTTAATAATA	52.5°C
ARL4C (CpG No. 8)	GGGACACCGCT GATCGTTTATAA TGTATTAATGGGT GATTTATGA	AAAACAAAACTAACCTCCCTATAT	CTATAAAAA ACCCATTCACT	52.5°C
ARL4C (promoter)	TTGGGGTTTGG AGGTTTTAGT	GGGACACCGCTGATCGTTTACAA CCTACCTAAAAAAATAAATCC	GGTTTGGA GGTTTTAGTTT	60°C

Primer sequences for bisulfate sequencing methylation analysis

Gene	Forward primer	Reverse primer
ARL4C (CpG No.6 and No.7)	GGGTGTAATTAA TTGTTAGTAATTG	GGGACACCGCTGATCGTTTAAAC CACTCCAAAATAATTTAATCT
ARL4C (CpG No. 8)	GGGACACCGCTGATCGTTTATA ATGTATTAATGGGTGATTTATGA	AAAACAAAACTA ACCTCCCTATAT

Supplementary Table S2: ARL4C DNA methylation level by bisulfite pyrosequencing analysis in lung SCCs

3'-UTR			Promoter region		
Specimen number	Tumor methylation level	Non-tumor methylation level	Specimen number	Tumor methylation level	Non-tumor methylation level
1	25.6±2.2	50.2±6	1	30.40±5.95	33.67
2	44.3±0.5	62.3±10	2	failure	93.15
3	32.3±2.1	50.3±4	3	23.93±3.55	41.57
4	41.1±2.4	not available	4	28.5±0.95	not available
5	37.8±1.7	52±4.2	5	failure	36.9
6	44.9±0.9	48.9±2.3	6	20.83±4.29	37.66
7	23.1±4.4	41.1±1.7	7	25.04±4.74	43.61
8	33.6±8.1	52±1.7	8	17.93±1.3	18.53
9	34.7±1.5	not available	9	26.34±9.19	not available
10	22.3±6	52.3±4.6	10	failure	44.5
11	35.6±3.7	59.1±5	11	26.38±9.91	25.42
12	26.5±6	not available	12	failure	not available
13	18.1±8.1	not available	13	failure	not available
14	failure	73.13	14	failure	17.16
15	38.1±7.8	not available	15	failure	not available
